# Alcohol, HMGB1, and Innate Immune Signaling in the Brain

**DOI:** 10.35946/arcr.v44.1.04

**Published:** 2024-08-08

**Authors:** Fulton T. Crews, Leon G. Coleman, Victoria A. Macht, Ryan P. Vetreno

**Affiliations:** 1 Bowles Center for Alcohol Studies, University of North Carolina School of Medicine, Chapel Hill, North Carolina; 2 Department of Pharmacology, University of North Carolina School of Medicine, Chapel Hill, North Carolina; 3 Department of Psychiatry, University of North Carolina School of Medicine, Chapel Hill, North Carolina

**Keywords:** alcohol, alcohol use disorder, microglia, cytokines, chemokines, HMGB1 protein, neuroinflammation, cholinergic neurons

## Abstract

**PURPOSE:**

Binge drinking (i.e., consuming enough alcohol to achieve a blood ethanol concentration of 80 mg/dL, approximately 4–5 drinks within 2 hours), particularly in early adolescence, can promote progressive increases in alcohol drinking and alcohol-related problems that develop into compulsive use in the chronic relapsing disease, alcohol use disorder (AUD). Over the past decade, neuroimmune signaling has been discovered to contribute to alcohol-induced changes in drinking, mood, and neurodegeneration. This review presents a mechanistic hypothesis supporting high mobility group box protein 1 (HMGB1) and Toll-like receptor (TLR) signaling as key elements of alcohol-induced neuroimmune signaling across glia and neurons, which shifts gene transcription and synapses, altering neuronal networks that contribute to the development of AUD. This hypothesis may help guide further research on prevention and treatment.

**SEARCH METHODS:**

The authors used the search terms “HMGB1 protein,” “alcohol,” and “brain” across PubMed, Scopus, and Embase to find articles published between 1991 and 2023.

**SEARCH RESULTS:**

The database search found 54 references in PubMed, 47 in Scopus, and 105 in Embase. A total of about 100 articles were included.

**DISCUSSION AND CONCLUSIONS:**

In the brain, immune signaling molecules play a role in normal development that differs from their functions in inflammation and the immune response, although cellular receptors and signaling are shared. In adults, pro-inflammatory signals have emerged as contributing to brain adaptation in stress, depression, AUD, and neurodegenerative diseases. HMGB1, a cytokine-like signaling protein released from activated cells, including neurons, is hypothesized to activate pro-inflammatory signals through TLRs that contribute to adaptations to binge and chronic heavy drinking. HMGB1 alone and in heteromers with other molecules activates TLRs and other immune receptors that spread signaling across neurons and glia. Both blood and brain levels of HMGB1 increase with ethanol exposure. In rats, an adolescent intermittent ethanol (AIE) binge drinking model persistently increases brain HMGB1 and its receptors; alters microglia, forebrain cholinergic neurons, and neuronal networks; and increases alcohol drinking and anxiety while disrupting cognition. Studies of human postmortem AUD brain have found elevated levels of HMGB1 and TLRs. These signals reduce cholinergic neurons, whereas microglia, the brain’s immune cells, are activated by binge drinking. Microglia regulate synapses through complement proteins that can change networks affected by AIE that increase drinking, contributing to risks for AUD. Anti-inflammatory drugs, exercise, cholinesterase inhibitors, and histone deacetylase epigenetic inhibitors prevent and reverse the AIE-induced pathology. Further, HMGB1 antagonists and other anti-inflammatory treatments may provide new therapies for alcohol misuse and AUD. Collectively, these findings suggest that restoring the innate immune signaling balance is central to recovering from alcohol-related pathology.

Alcohol intoxication alters neuronal networks that markedly impact impulsiveness, balance, and other important brain functions. Although acute intoxication has immediate dangers, alcohol use disorder (AUD) has a lasting impact on individuals and families. AUD is considered a chronic relapsing disease linked to cycles of intoxication often initiated in adolescence that change neuronal networks and personality. The neurobiology of AUD, and substance use disorder (SUD) in general, is conceptualized as first involving attention–reward circuits, with each cycle of intoxication and withdrawal progressively increasing anxiety-like behaviors, hyperkatifeia, and disruption of frontal cortical executive function that inhibits impulsiveness.^1^ Emerging studies on brain physiology using magnetic resonance imaging (MRI) in humans have identified neuronal networks that include visual, sensorimotor, auditory, and salience networks; frontal cortical–executive impulse inhibition; and brain default mode networks. Multiple studies support plasticity-induced changes in neuronal networks related to drug-induced craving, impulsivity, and other factors that contribute to alcohol drinking and symptoms related to AUD.^1^ The mechanisms that alter neuronal networks in AUD and SUD have been linked to increases in neuroimmune gene expression as well as other genes.

## Neuroimmune Signaling

Neuroimmune signaling generally refers to increases in pro-inflammatory gene expression in neurons and other brain cells. The presence of pro-inflammatory signaling is surprising since there are rarely pathogens in the brain, suggesting other functions for these processes in the brain. Pro-inflammatory signaling has emerged as an important mechanism in adaptations of neuronal networks that impact drinking as well as brain responses to alcohol and alcohol cues. Several previous reviews have summarized neuroimmune regulation of alcohol drinking,^2^,^3^ clinical trials of anti-inflammatory drugs for treatment of AUD,^4^ and similarities of neuroimmune signaling in AUD and Alzheimer’s disease.^5^ This review discusses findings related to alcohol and pro-inflammatory gene expression that support the hypothesis that pro-inflammatory signals alter brain cellular transcriptomes, changing the neuronal networks that underlie AUD. Although clinical and preclinical studies are finding promising new therapeutic targets for AUD, pro-inflammatory signaling is complex and its functions in the brain are poorly understood. Studies suggest that changes in gene transcription induced by pro-inflammatory signaling are reversible,^6^ making them a particularly promising prospect for new treatments. This review proposes high mobility group box protein 1 (HMGB1), a specific pro-inflammatory cytokine-like protein expressed in all brain cells, as a potential therapeutic target due to its broad pro-inflammatory receptor actions. Further, studies on the mechanisms of adolescent binge drinking–induced changes in the adult brain are highlighted to illustrate how preventing the pro-inflammatory response to alcohol during initiation of drinking will reduce lifelong risks for AUD and problems related to alcohol drinking. Understanding mechanisms of AUD and pro-inflammatory signaling changes in the brain will aid prevention and treatment efforts.

Historically, the brain has been considered “immune privileged” because antibodies and the T and B lymphocytes that mediate adaptive immunity are generally not found in the brain.^7^ Brain cells do express cytokines, chemokines, their receptors, as well as the complement cascade genes, oxidases, and proteases associated with innate immune monocyte-macrophage responses to “acute phase infection-trauma.”^8^ However, functions of pro-inflammatory genes in the brain can differ from systemic responses. Contrary to initial assumptions that immunological memory only exists in the lymphocytes of the adaptive immune system, which are not generally found in the brain, recent evidence suggests that microglia and other monocyte-macrophages adapt to stimuli, becoming sensitized, or primed, with an innate immune memory.^9^–^11^ These adaptations may occur with cycles of alcohol exposure.^12^ Although neuroimmune signaling crosses all brain cell types, microglia are the resident brain monocyte-like cells. These unique brain cells contribute to cellular and synaptic development and integration into functional neurocircuits.

### Toll-like Receptors

In healthy brains, expression of pro-inflammatory Toll-like receptors (TLRs), cytokines, and chemokines is low and occurs primarily in microglia, with transient changes in expression linked to development and synaptic physiology. Signaling between neurons and microglia increases expression of interleukin (IL) 4 and IL-10, two anti-inflammatory cytokines^13^ that can inhibit induction of TLRs and other brain pro-inflammatory genes. Although initially discovered as receptors for pathogens, TLRs have been found to respond to endogenous agonists such as HMGB1 that are not related to infections.^6^,^14^ Recent findings have also revealed that astrocyte and neuron activation induces TLRs and other innate immune signaling receptors that can contribute to the initiation and propagation of innate immune responses.^15^–^17^ Pro-inflammatory signaling has emerged as a key mechanism contributing to brain responses to alcohol, and progressive changes during cycles of alcohol exposure may contribute to the development of AUD. Clinical and preclinical studies are currently investigating microglial and brain pro-inflammatory antagonists and offer great promise for improved AUD treatments.^4^ These findings suggest that AUD changes in brain networks and behavior involve HMGB1, TLRs, and other innate immune signaling molecules.

### HMGB1

HMGB1 is emerging as a key signal in the brain. HMGB1 is a relatively small nuclear protein with high expression in neurons that is released in acute response to traumatic injury, stress, and/or infections,^18^ activating innate immune signaling.^6^,^19^ Innate immune signals are characterized by feed-forward cellular activation across neuronal and glial cells, increasing expression of cytokines and their receptors, including TLRs. Emerging studies in the brain indicate that HMGB1 alone or in heteromers with endogenous molecules like IL-1-beta^20^ and nucleic acids can activate multiple TLRs.^6^,^14^,^19^,^21^ Although acute pro-inflammatory responses amplify within and across cells, specific cytokine signals change over time through complex mechanisms not well understood. Traumatic injury initiates pro-inflammatory signaling that eventually subsides and transitions to growth signals that stimulate cell proliferation, regrowth, and tissue healing.^22^ This progressive change from pro-inflammatory signals to healing factors, anti-inflammatory cytokines, and other signals that promote wound healing is orchestrated by tissue-specific, monocyte-like cells. It is not known how microglia in the brain fit into this generalized monocyte wound-healing dogma, but microglia are known to release both pro-inflammatory and trophic signals.^23^

HMGB1 functions in the nucleus are not well characterized; however, in general, HMGB1 can bend DNA and enhances binding of transcription factors such as p53^24^,^25^ and estrogen receptor^26^ to DNA. The role of HMGB1 in the immune system, both nuclear functions and pro-inflammatory signaling, has been recently reviewed.^27^ This review focuses on HMGB1, alcohol, neuroimmune signaling in the brain, and persistent changes in the brain due to adolescent binge drinking that overlap with those found in the brains of people with AUD.

This review first describes aspects of innate immune signaling in the central nervous system (i.e., neuroimmune signaling), with a focus on microglia; the impact of innate immune signaling on synaptic circuitry; and the role of HMGB1 activation of TLR pathways. It then focuses on emerging studies on how alcohol impacts neuroimmune signals across microglia, other glia, and neurons through epigenetic changes in gene transcription that alter synapses, neurocircuitry, and neuronal networks that contribute to the development of AUD. Both preclinical and postmortem human brain studies will be presented, revealing new hypothetical mechanisms on development of AUD. There is a large number of neuroimmune genes, including pro-inflammatory cytokines, chemokines, complement, proteases, and receptors. A detailed review of all these signals in the brain is beyond the scope of this article. This review focuses on HMGB1 and TLR pathways that have been linked to alcohol drinking and AUD and attempts to integrate human and preclinical studies.

## Search Methods and Results

Using the search terms “HMGB1 protein,” “alcohol,” and “brain,” the authors found 54 references in PubMed, 47 in Scopus, and 105 in Embase, for articles published between January 1991 and November 2022; the earliest article found was published in 2007. Searching PubMed for “brain” alone resulted in about 2 million papers, “alcohol-ethanol” alone found about 200,000 papers, and “HMGB1” identified around 10,000 papers; combining the search terms reduced the number of papers to about 50. Five articles not written in English were excluded, for a total of about 100 articles that are included in this review.

## Results of the Reviewed Studies

### Innate Immune Signaling and Synaptic Circuitry

Increasing evidence suggests that the neuroimmune system is involved in brain function, regulating synapses and excitability as well as elements of learning and memory. For example, low concentrations of the key pro-inflammatory cytokine tumor necrosis factor alpha (TNF-alpha) have been found to be required for long-term potentiation—an adaptive brain synapse response and potential mechanism of learning.^28^ Conversely, high levels of TNF-alpha disrupt long-term potentiation in the hippocampus.^29^ These findings suggest that transient changes in specific brain pro-inflammatory signals contribute to normal functions and development that are not related to immune responses and do not activate the spread of pro-inflammatory signals.

Studies in this century have led to the discovery that neuroimmune signaling is persistently increased in most brain diseases, including AUD as well as other psychiatric and neurological diseases.^6^,^12^,^14^,^18^,^23^ The relationship is not clear in part because there are many pro-inflammatory cytokines, and for practical reasons, studies need to focus on just a few assumed to be representative of increased neuroimmune signaling. This complicates understanding of alcohol-induced changes, especially because those changes are dependent upon the amount of alcohol consumed. Most studies of individuals with AUD and AUD models involve binge drinking and even extreme binge drinking. Binge drinking is defined as consuming enough alcohol to become intoxicated, which is defined by a blood ethanol concentration of 80 mg/dL, or approximately 4–5 drinks within 2 hours. Extreme binge drinking, or high-intensity drinking, is defined as consuming at least twice as much (i.e., 10–15+ drinks) per occasion. One study comparing non-binge and binge drinkers’ responses to pictures of alcohol drinks found binge drinkers had increased craving for alcohol, as well as increased blood levels of the pro-inflammatory cytokine IL-6 and greater prefrontal cortex activation.^30^ These observations are consistent with binge drinking sensitizing alcohol cues and cytokine responses to cues.

HMGB1 it is a broad-acting pro-inflammatory protein that can act on TLRs; it is induced by ethanol in preclinical studies and increased in postmortem AUD brain with positive correlations to lifetime alcohol consumption.^31^ In a study of Spanish university graduate students, men and women who binge drank, compared to controls, had increased activation of monocyte TLR4 and nuclear factor kappa-light-chain-enhancer of activated B cells (NF-kappa-B) as well as greater pro-inflammatory cytokine/chemokine release, oxidative stress, and lipid peroxidation that correlated with blood ethanol estimates. Moreover, females who binge drank had increased HMGB1 blood levels and other pro-inflammatory markers that correlated with worse scores on episodic memory and executive functioning tasks.^32^ As HMGB1 crosses the blood–brain barrier, changes in the blood likely reflect changes in the brain. These findings are consistent with the hypothesis that binge-drinking–induced increases in HMGB1 contribute to priming and sensitization of pro-inflammatory responses. Models of rat adolescent binge drinking have found persistent increases in microglial CD11b, a marker of microglial and monocyte priming, suggesting that cycles of binge drinking lead to persistent increases in pro-inflammatory gene expression.^6^,^10^,^33^

Binge-drinking–induced increases in HMGB1 induce pro-inflammatory responses that also change neuronal excitability and alter neuronal networks, with cycles of binge drinking leading to progressive adaptations. Hippocampal epilepsy seizures provide an example of how repeated HMGB1 increases alter neuronal networks. An initial seizure releases and increases expression of neuronal HMGB1 and IL-1-beta that persist; this increased expression sensitizes neurons to excitation, lowers seizure threshold, and increases risks of future seizures with each additional seizure.^34^ Similarly, cycles of chronic ethanol drinking increase HMGB1 in the amygdala, sensitizing withdrawal anxiety that is inhibited when HMGB1 is blocked by antagonists.^35^ Glutamate excitation also releases HMGB1 from hippocampal slices,^36^ and activation of pain sensory neurons releases HMGB1, triggering pain.^37^ Similarly, in models of depression-like behaviors induced by repeated social defeat stress, prefrontal cortical HMGB1 is increased and knockdown or antibody antagonists block depression-like responses.^38^ Thus, in the brain, HMGB1-related neuroimmune signaling contributes to ethanol and stress responses that can increase neuronal excitability and change networks. There are extensive studies on brain regional changes related to the development of AUD; however, how each region’s neuronal and glial changes are related to HMGB1 or other innate immune signals and the network dysregulation that contributes to progression to AUD is poorly understood. Identification of key innate immune signals contributing to AUD will provide new targets for therapies.^14^

### Toll-like Receptors and HMGB1

TLRs were first discovered as innate immune receptors sensing bacteria and viruses as well as trauma-induced cellular damage signals. TLRs belong to the Toll/interleukin-1 receptor superfamily of single membrane-spanning proteins that share a Toll/interleukin-1 receptor intracellular domain, which mediates protein-protein signaling.^39^ Endosomal TLRs (i.e., TLR3, TLR7, and TLR9) respond to DNA and RNA. In general, these receptors utilize the myeloid differentiation primary response 88 (MyD88) adapter protein complex, which activates multiple kinases as well as downstream NF-kappa-B and activated protein-1 (AP-1) transcription factors. These key transcription factors subsequently induce the expression of several pro-inflammatory cytokines, receptors, and other mediators to spread the response. There are at least 10 TLRs in humans and 12 in mice.^40^ TLRs have multiple endogenous ligands, including HMGB1, which creates “sterile inflammation”—a pro-inflammatory response in the absence of an invading organism. However, TLR signaling pathways in brain cells are not well understood as most signaling has been characterized in peripheral immune cells. Emerging studies on increased brain TLR expression in people who commit suicide or have depression, neurodegeneration, or SUD without evidence of infections and the discovery of endogenous agonists such as HMGB1 suggest that TLRs have a role in brain physiology and pathology. Overall, TLR expression is low in healthy brain. Microglia express low levels of multiple TLRs, but TLR3 is enriched in astrocytes^41^ and TLR4 is expressed ubiquitously by neurons and glia, although at much lower levels than HMGB1.^6^

TLR signaling through NF-kappa-B in neurons is complex,^42^ although some studies have linked it to synaptic plasticity, learning and memory,^43^ as well as neuroinflammation and neurodegeneration.^6^,^14^ The effects of activation of specific TLRs may vary between cell types and across various brain regions with changing microenvironments; these differences need further investigation. HMGB1 alone or in heteromers with cytokines, chemokines, DNA, and RNA can contribute to activation of all TLRs.^44^,^45^ TLRs within endosomes (i.e., TLR3, TLR7, TLR8, and TLR9) respond to RNA and DNA. These TLRs were initially thought to respond exclusively to RNA and DNA; however, HMGB1/nucleic acid heteromers have been discovered to chaperone RNA and DNA into cells contributing to endosomal TLR activation.^14^,^21^,^44^ Immune cells lacking HMGB1 lose endosomal TLR3 and TLR7 activation of interferon signaling, suggesting HMGB1 is a required nucleic acid sensor^46^ important for TLR3 activation.^47^ More recent studies have discovered ethanol induces release of extracellular vesicles from microglia containing HMGB1/let-7 microRNA (miRNA) heteromers that activate neuronal TLR7.^21^ Also, receptor for advanced glycation end products (RAGE), an HMGB1 receptor, contributes to cellular uptake and endosomal TLR activation.^48^,^49^ Whereas most TLRs share signaling through the signal-activating protein MyD88, TLR3 recruits a different protein, TRIF; however, more studies are needed to understand these signals in the brain.^14^

Although infections can trigger immune signaling changes in the brain that affect behavior (e.g., mood, appetite), TLR signaling in the brain likely is associated with more than brain inflammation. A number of studies have linked activation of TLR3 to ethanol drinking, including escalation of drinking in mice^50^ and alcohol self-administration in rats.^51^,^52^ In contrast, transgenic male mice lacking TLR3 drank less alcohol and required more time to recover from ethanol sedation.^53^ Transgenic mouse and ethanol transcriptome studies, conducted primarily by the Integrative Neuroscience Initiative on Alcoholism’s Neuroimmune consortium, led to a focus on astrocyte TLR3^54^ as regulating ethanol drinking.^55^ Studies in rats found that operant alcohol self-administration (i.e., rats had to press a lever to get a drink) was increased by TLR3 agonists^51^,^52^ as well as TLR7 agonists.^56^ Additionally, TLR4 levels were increased in alcohol-preferring rats, and inhibition of TLR4 decreased binge drinking.^57^ These effects were linked to interactions between gamma-aminobutyric acid subtype A (GABAA) receptor and TLR4.^58^ Other studies suggested that brain TLR signaling was involved in development and neurogenesis, as well as neurocircuitry, mood, and cognition,^59^,^60^ all of which are altered in AUD. TLRs should be considered as therapeutic targets for AUD.

**Figure 1:**
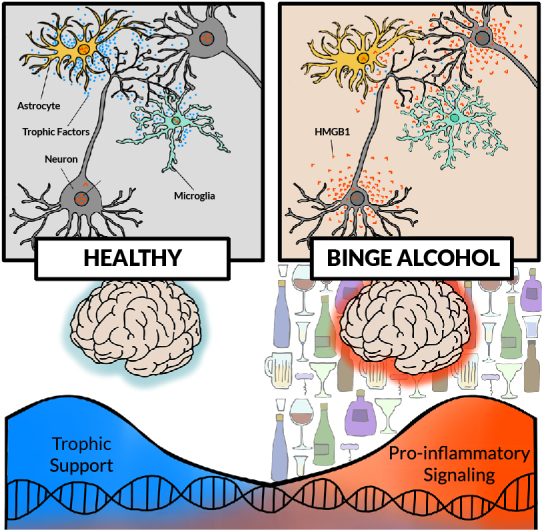
Effects of chronic alcohol activation of pro-inflammatory signaling in the brain (Top left) In a “healthy” brain, neurons, astrocytes, and microglia release growth signals (i.e., trophic factors such as brain-derived neurotrophic factor [blue dots]) supporting neuronal and glial functions. (Top right) With chronic binge alcohol exposure, all cells show changes, such as reductions in soma and synapses. Other changes include increased high mobility group box protein 1 (HMGB1) release (orange dots) in neurons, hyper-ramification and pro-inflammatory activation of microglia, and activation of astrocytes. (Bottom) Without alcohol exposure, transcription of growth factors, such as brain-derived neurotrophic factor, is high, providing trophic support. In a brain exposed to chronic binge drinking, cellular transcription shifts, with increasing pro-inflammatory transcripts. *Note:* Not shown are other pro-inflammatory signals, although pro-inflammatory changes in transcription likely involve multiple signals; all brain cell types are proposed to change cellular transcriptomes and subtypes.

A characteristic of pro-inflammatory TLR responses is amplification of signals across cells through induction of TLRs, cytokines, chemokines, interferons, and their receptors (Figure 1). The spread of pro-inflammatory signaling is exemplified by HMGB1, which induces production of additional cytokines and their receptors in adjacent cells (Figure 1).^12^,^37^ HMGB1 is a nuclear protein that in the brain may have DNA protective functions.^61^,^62^ Following acetylation, HMGB1 translocates from the nucleus to the cytoplasm, where it is packaged in vesicles for release. HMGB1 release by ethanol results in reduced nuclear HMGB1 content. Released HMGB1 can bind directly to TLR4 or RAGE.^63^ HMGB1 also activates other TLRs as well as cytokine and chemokine receptors as heteromers. This HMGB1 pro-inflammatory signaling is involved in hyperexcitable states, such as ischemic^64^ and epilepsy-induced seizures,^65^ through increased glutamatergic signaling. Studies in mice found that sleep deprivation lead to neuronal HMGB1 release and synaptic metabolic stress.^66^ In hippocampal brain slice culture, glutamate excitation released HMGB1, with high levels of glutamate causing neurotoxicity, which was reduced by the HMGB1 antagonist glycyrrhizin.^36^ Histone deacetylase inhibitors also released HMGB1 from hippocampal brain slices, consistent with nuclear acetylation stimulating release.^36^ Thus, HMGB1 release amplifies signaling through induction of its own receptors.

HMGB1 can activate TLR4 and RAGE receptors alone as well as in heteromers with cytokines or chemokines. One example of an HMGB1 heteromer is the HMGB1/IL-1-beta heteromer, which is more potent at the IL-1 receptor than IL-1-beta alone.^20^ HMGB1 heteromeric complexes are particularly dynamic, forming with cytokines, DNA, or RNA to activate TLR receptors, as well as increasing expression of IL-6, interferon,^20^ and likely many others not assessed. HMGB1 and HMGB1 heteromer complexes can activate essentially all TLRs,^44^ contributing to HMGB1 as a pro-inflammatory signal.

Chronic binge-like ethanol exposure of rodents increases brain HMGB1 levels similar to levels found in the postmortem brains of humans with AUD.^15^ Alcohol drinking in rats increases HMGB1 only after several drinking episodes. Multiple drinking cycles increase HMGB1 as well as promoting anxiety-like behaviors. Treatment with corticotropin-releasing factor type 1 receptor (CRF1R) and HMGB1 antagonists restores chronic alcohol-induced anxiety-like behavior.^35^ It is not clear what forms of HMGB1 are released from neurons; they likely include acetylated HMGB1, HMGB1 heteromers, as well as vesicular and free HMGB1. However, HMGB1 clearly is a key innate immune signaling molecule with multiple forms in the brain that are impacted by cycles of alcohol drinking.

### HMGB1 and Extracellular Vesicles

Emerging studies have discovered that HMGB1 and other signals within extracellular vesicles (EVs) contribute to innate immune signaling. EVs released from cells include exosomes (40–100 nanometer diameter) and microvesicles (0.1–1.0 micrometer diameter), which contain many different proteins, small micro-RNAs (miRNAs) that are not made into proteins, and lipid cargo.^67^ For example, TLR7, an endosomal receptor for single-stranded RNA viruses, is activated by EVs containing the endogenous miRNA let-7b, which can result in neuronal death.^21^,^68^ In brain slice culture, ethanol increased the secretion of EVs containing miRNA let-7b and shifted the binding of let-7b from its chaperone Argonaute to HMGB1. As a result of this switch, let-7b is released in vesicles that activate TLR7 receptors rather than being trafficked to miRNA targets, resulting in TLR7-mediated neurodegeneration.^21^ Let-7 is increased in postmortem brain samples from humans with AUD and in rodents with chronic ethanol exposure.^69^ Indeed, HMGB1 is critical for immune responses of each of the endosomal TLRs (i.e., TLR3, TLR7, and TLR9).^46^ Ethanol also induces the release of EVs that induce pro-inflammatory cytokines TNF-alpha and IL-1-beta when applied to naive brain slice cultures. Microglial depletion, blockade of EV release, and the HMGB1 antagonist glycyrrhizin all block EV induction of TNF-alpha and IL-1-beta in response to ethanol, consistent with EVs contributing to ethanol-induced HMGB1 pro-inflammatory signaling.^70^ Thus, HMGB1 and EV release from microglia represents a newly discovered component of ethanol-activated innate immune signaling that may contribute to AUD and warrants further investigation.

### Microglia, the Innate Immune Cells in the Brain

Early in embryonic development, monocyte progenitor cells migrate to developing tissues where they contribute to tissue maturation and become resident tissue-specific monocytes.^71^ In mice, yolk sac progenitors migrate to the brain early in embryonic development, prior to mesodermal expansion of progenitors migrating to the lung and then the liver.^72^ In the liver, progenitors become resident Kupffer cells. In contrast, in the brain, these progenitors become microglia—unique brain-specific, monocyte-like cells.^73^ These microglial progenitors develop along with the brain and mature in parallel with maturation of neural circuitry.^74^,^75^

Monocyte-like cells have tissue-specific functions, although they share basal expression of innate immune genes. All macrophage/monocyte-like cells also share changes in functional phenotype that have been categorized as pro-inflammatory, phagocytic, and/or trophic growth-stimulating forms and that are regulated by epigenetic mechanisms.^76^,^77^ Microglia can transition between these “resting” states and activated phenotypes in response to infections, stressors, and drugs of abuse, such as alcohol and cocaine.^21^,^78^–^80^ Efforts to clearly define a microglial pro-inflammatory state (often referred to as M1) have been confounded by the diversity of microglial transcription states, with single-cell transcriptome studies defining five to 10 common expression profiles that confound simple definitions. Across states of activation, microglia can produce pro-inflammatory cytokines (e.g., TNF-alpha, IL-1-beta) as well as inducible nitric oxide synthase (iNOS) and nicotinamide adenine dinucleotide phosphate (NADPH) oxidase, contributing to the propagation of the pro-inflammatory response in the brain.

Microglia are long-lived cells, and each microglia has its own spatial domain wherein it maintains the local environment. Unlike neurons, astrocytes, or myelinating cells that are cemented together with the extracellular matrix, microglia move around and phagocytize waste, stimulate synapse formation, remove synapses, and/or regulate myelin. The healthy brain has a stable density of microglia that varies across brain regions and with changes in the local environment.^23^ Endogenous microglial progenitors divide to maintain levels throughout the lifespan, with aging associated with progressive increases in expression of microglial genes.^60^ Microglia differentiate into diverse subtypes that include trophic, pro-inflammatory, engorged, and inactivated dystrophic morphologies that have subtle differences and are poorly linked to diverse microglial subtype transcriptomes. Microglial signals in the brain generally have protective neuron- and brain-specific functions. Complement proteins in the blood—which are derived from numerous cells, including microglia—help identify infectious agents, clear antibody complexes, and clot blood, whereas in the brain, microglial complement type 1 interacts with complement component 3 (C3) on synapses to signal and facilitate microglial phagocytosis of synapses.^81^ During development, microglia, astrocytes, oligodendrocytes, and progenitors, along with growing neurons, coordinate to form focused, functional mature neurocircuits, with dysfunctional neurons and inactive synapses removed by microglia.^82^

The role of microglia in neurocircuitry has been studied using molecular techniques in mice that deplete microglia from the brain. Microglial depletion or selective depletion of the microglial chemokine receptor CX3CR1 results in increases in synapses as well as increased complement C3, the synaptic “eat me signal.”^83^,^84^ Generally, microglia repair and support neurons through specific microglial-neuron signals promoting growth and inhibiting pro-inflammatory signals. For example, fractalkine (i.e., CX3CL1) is a chemokine produced and released by neurons that acts on microglial CX3CR1, inhibiting pro-inflammatory activation.^85^ In the hippocampus, CX3CL1 is upregulated briefly during discrete temporal learning windows, consistent with a synaptic protective process that allows synaptic scaling and memory formation.^86^ Thus, while microglia and neuroimmune signaling typically contribute to healthy brain development and function, disruptions in microglial and innate immune signaling can also disrupt this process, leading to downstream consequences on cognition and behavior.

Pro-inflammatory genes are induced by alcohol in the brain, which is consistent with microglial pro-inflammatory priming or sensitization by alcohol exposure; however, the impact of alcohol on microglial synapse phagocytosis and microglial trophic support functions is poorly understood. The impact of alcohol on microglia differs with dose, time, and brain region.^5^,^6^,^33^,^87^ Microglial pro-inflammatory signaling and HMGB1 likely contribute to cycles of chronic binge drinking leading to persistent pro-inflammatory TLR and other gene induction in astrocytes and neurons that changes neuronal networks. Additional cell biological and morphological studies on microglia are needed to clearly understand the impact of alcohol-induced changes in brain microglia. However, studies support a role for microglia in brain development, particularly development of synapses, neurocircuits, and neuronal networks, which are disrupted by binge drinking–induced pro-inflammatory cytokines that underlie the neurobiology of AUD.

### Acute Binge Drinking and Microglial Priming

Alcohol drinking varies widely, although individuals with AUD and certain groups of adolescents exhibit binge drinking and extreme binge drinking more often than older adults. Animal studies are needed to understand the mechanisms of how binge and extreme binge drinking impact the brain. A central hypothesis of the mechanisms of progression from binge drinking to AUD is that cycles of intoxication and/or stress promote transitions from binge drinking/intoxication to withdrawal and negative affect, to craving and preoccupation, to repeated binge drinking, and finally to AUD.^88^ These stages are related to progressive changes in neurocircuitry that contribute to loss of executive functions such as goal setting and impulse control, with increasing negative mood and reward seeking. Neuroimmune signaling, particularly in relation to microglial phenotypic changes, has been proposed to contribute to these changes in neurocircuitry.^12^,^89^–^91^

**Figure 2:**
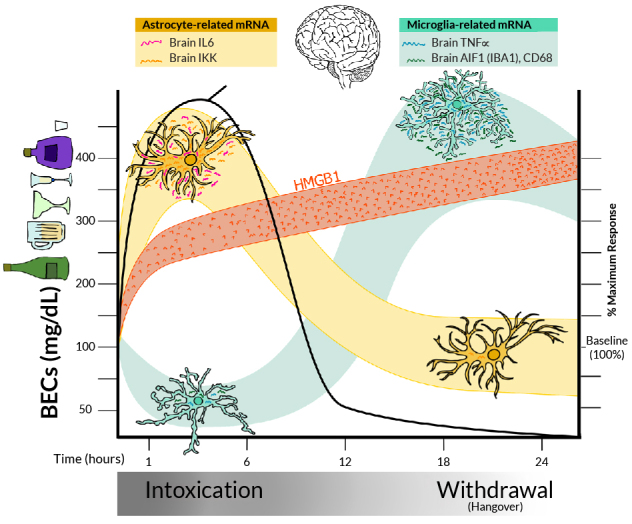
Model of acute binge-drinking–induced changes in pro-inflammatory signaling and other changes in the rat brain After administration of an acute binge-drinking dose of ethanol, BECs (black line) rise rapidly within the first 6 hours, indicating intoxication, before declining again. Later timepoints (18–24 hours), when BECs return to zero, reflect acute withdrawal.^20^,^95^ Pro-inflammatory signaling responses include HMGB1 (orange), astrocyte (yellow), and microglial (green) responses. All three brain cell types (neurons, microglia, and astrocytes) show ethanol-induced HMGB1 release, resulting in rapid HMGB1 increases in blood and brain during intoxication that persist into acute withdrawal.^21^,^36^,^60^ Other pro-inflammatory cytokines in the brain show different patterns in response to alcohol. Levels of IL-6 and IKK, which are released by astrocytes, increase during intoxication, consistent with astrocyte pro-inflammatory activation, before declining again. In contrast, microglial markers, such as TNF-alpha, decrease during intoxication but increase during withdrawal from an acute binge.^33^ Thus, pro-inflammatory cytokines are increased during both intoxication and acute withdrawal, but responses are not homogeneous, highlighting both the importance of specifically determining gene responses and the difficulty in generalizing findings from a single pro-inflammatory marker as representative of all pro-inflammatory cytokines. *Note:* Aif1/Iba1, allograft inflammatory factor 1/ionized calcium-binding adapter molecule 1; BEC, blood ethanol concentration; HMGB1, high mobility group box protein 1; IKK, I-kappa-B kinase; IL-6, interleukin-6; mRNA, messenger RNA; TNF-alpha, tissue necrosis factor alpha.

Consistent with ethanol binge drinking and stress increasing microglial pro-inflammatory responses in the brain, removal of brain microglia through molecular techniques results in a loss of acute ethanol induction of pro-inflammatory genes.^31^,^33^,^92^ For example, in mice, acute restraint stress and acute binge drinking doses of ethanol increase CD11b+-activated “primed” microglia in the prefrontal cortex and nucleus accumbens,^33^ two brain regions involved in decisions and reward respectively. Acute ethanol induction of neuroimmune genes illustrates the complexity of neuroimmune signaling and the challenges of translating cellular to whole-brain studies. In studies of rats with acute binge drinking, mRNA of the pro-inflammatory markers IL-6 and I-kappa-B-alpha (a marker used to assess immune activation through increased NF-kappa-B transcription) increased in the brain within 3 hours, but pro-inflammatory TNF-alpha mRNA was decreased at that time.^93^,^94^ Similar studies in mice found that acute binge ethanol treatment reduced TNF-alpha mRNA during intoxication, around 3 hours after the initial dose, but that levels increased during acute withdrawal 17 hours after the initial binge dose (Figure 2).^95^ As models of immune signaling generally suggest that all pro-inflammatory genes should increase during acute alcohol exposure, the concurrent increase of IL-6 mRNA and decrease of TNF-alpha mRNA during intoxication are confounding and appear contradictory. Further, it is unclear how the initial decrease in TNF-alpha mRNA during intoxication later reverses to an increase during acute withdrawal. One explanation is that multiple cells respond to ethanol in the brain. Studies removing microglia using depletion protocols find that the delayed TNF-alpha induction after acute ethanol treatment, but not the IL-6 response, is lost in the microglial-depleted hippocampus. This is consistent with differential cellular pro-inflammatory responses to ethanol that vary across time, across cell types, and with blood ethanol concentrations (Figure 2).

This example illustrates the complexity of neuroimmune responses and the problems with assessing individual cytokines as representative of general neuroimmune signaling. Further, rat studies have suggested that acute ethanol-induced increases in plasma cortisol contribute to cytokine changes, further complicating mechanisms.^94^,^96^ Other studies found acute ethanol-induced reductions in brain-derived neurotrophic factor (BDNF) in males, with increased IL-6 and I-kappa-B-alpha mRNA in the amygdala and the hippocampus.^93^ Reduced BDNF trophic factor expression and increased proinflammatory gene expression are linked to shifts in transcription across multiple brain cell types (see Figure 1). Episodes of binge drinking, similar to repeated chronic restraint stress, persistently activate microglia across stress-responsive brain regions.^97^ Alcohol is a pharmacological stressor, releasing HMGB1 and activating pro-inflammatory gene induction.

Increases in microglial activation are frequently associated with increases in brain pro-inflammatory cytokines, including TNF-alpha and IL-1-beta, which have been linked to increased risks for mental disease and mood disorders.^98^,^99^ Furthermore, both stress and acute ethanol increase HMGB1 in the rat hippocampus, priming microglia and increasing IL-1-beta synthesis.^92^ Microglia express specific proteins that help define their function, such as Iba1 and CD11b, which increase with TNF-alpha induction, and the lysosomal protein CD68 that suggests increased phagocytosis. Acute ethanol dose-response analyses in mice found that only high binge drinking doses acutely impacted TNF-alpha and microglial markers, suggesting that the effect of alcohol on microglial activation is dose-dependent.^33^,^93^ Binge drinking ethanol exposure induces a biphasic response (see Figure 2). Early time points associated with high blood ethanol levels show reduced brain expression of Iba1, CD11b, CD68, and TNF-alpha.^95^ This is consistent with studies finding that lipopolysaccharide (LPS) induction of TNF-alpha and IL-1-beta in monocytes is blunted during acute alcohol exposure, coupled with reduced NF-kappa-B activation.^100^ Thus, binge drinking levels of ethanol inhibit monocyte and microglial activation during intoxication, even though HMGB1, an endogenous TLR agonist, is released by ethanol. Other studies that reported reduced TNF-alpha mRNA during binge intoxication found increased IL-6 and IKK-beta, a marker of pro-inflammatory NF-kappa-B signaling.^33^,^93^ These findings are paradoxical because pro-inflammatory cytokines generally increase expression of themselves and other cytokines. Astrocyte-like cells in ethanol-treated cultures showed HMGB1 release and ethanol induction of pro-inflammatory cytokine IL-6, consistent with different pro-inflammatory gene responses across brain cells that require more detailed studies to clearly understand aspects of the acute alcohol response and adaptations that occur with chronic cycles of binge drinking (see Figure 2 and legend).

As mentioned above, microglia are blocked from activation early in intoxication, but as ethanol is eliminated after several hours, there is a robust increase in pro-inflammatory TNF-alpha and CCL2 mRNA as well as microglial activation markers CD68 and Iba1 mRNA in the brain, consistent with microglial activation.^95^ The increase in cytokines following alcohol drinking contributes to acute withdrawal with hangover sickness-like symptoms. Each acute alcohol intoxication and withdrawal experience increases microglial TNF-alpha that sensitizes cells for future activation, a process known as “microglial priming.” Thus, the acute binge-drinking–induced increase in TNF-alpha and microglial activation markers is consistent with initiating priming, perhaps a form of innate immune memory that could be related to binge-drinking–induced increases in HMGB1. Microglial priming is related to the development of chronic neurodegeneration diseases.^101^ Ethanol treatment of mice for 10 days with binge drinking doses of ethanol increased brain TLR mRNA59 and subsequently the response to TLR agonists (e.g., LPS-TLR4^102^ and PolyIC-TLR3^103^), consistent with ethanol exposure inducing sensitization to pro-inflammatory TLR activation. Microglial activation can result in a variety of phenotypes that are only beginning to be understood. Microglia contribute to the local cellular milieu, responding to and impacting astrocytes and neurons that vary across brain regions. Microglial priming is linked to long-lasting changes in gene expression through histone and DNA modifications that epigenetically repress or enhance transcription of pro-inflammatory genes.^33^,^76^,^87^ Increases in microglial expression of HMGB1, Iba1, CD11b, and TNF-alpha by a single exposure poise these genes for induction that over time leads to progressive increases with each cycle of activation. Although chronic binge drinking models and AUD increase many pro-inflammatory genes in the brain, the cell types involved and microglial phenotype shifts are not fully understood. Interestingly, one study using a binge-like alcohol exposure model suggested that the activated microglial morphology progressed during chronic exposure to a dystrophic, senescent-like reduced function phenotype.^104^ Taken together, these findings are consistent with acute binge drinking inducing pro-inflammatory gene induction, microglial priming, and progressive changes in microglial phenotype, which can disrupt neuronal networks contributing to development of negative mood, cognitive deficits, and progression toward AUD.

### HMGB1 Signaling in AUD and Rodent Models

Analysis of innate immune signaling of postmortem human brain provides insight on AUD-induced changes in the brain. HMGB1, TLR, and other neuroimmune genes as well as microglial activation markers are increased in postmortem AUD brain,^6^,^15^ and chronic ethanol treatment of rodents causes similar persistent neuroimmune activation.^105^ Further, HMGB1 and its receptors RAGE, TLR2, TLR3, TLR4, and TLR7 are all increased in the orbital frontal cortex and the hippocampus in human postmortem tissue, suggesting persistent upregulation of innate immune signaling cascades.^15^ Although the postmortem AUD brain shows increases in nuclear HMGB1 based on immunohistochemical staining, Western blot analysis, and mRNA PCR in the prefrontal cortex^15^ and the hippocampus,^21^,^106^ global transcriptome studies in humans,^55^ mice,^107^ and rats^108^ do not find HMGB1 mRNA as an alcohol-induced mRNA. The data on humans, rats, and mice showing alcohol-induced increases in HMGB1 protein suggest that HMGB1 regulation occurs primarily at the protein level. This is consistent with HMGB1 having diverse nuclear and cytokine-like functions that are mainly controlled at the protein level. Recent single-cell transcriptome studies on prefrontal cortex of people with AUD found HMGB1 mRNA in all brain cell types, with some enrichment in astrocytes.^55^ Although changes in HMGB1 mRNA were not detected in the brains of individuals with AUD, high mRNA levels in astrocytes are associated with increases in interferon gene expression.

HMGB1 has an additional complexity because it signals through protein heteromers (i.e., complexes of HMGB1 and a cytokine, DNA, or RNA) that enhance pro-inflammatory signaling. HMGB1 protein is increased in the AUD brain in the hippocampus and studies have found increased levels of HMGB1/IL-1-beta heteromers,^20^ as well as HMGB1/let-7 miRNA heteromers,^21^,^106^ in postmortem human AUD brain. These findings suggest that HMGB1 can activate pro-inflammatory signaling through multiple receptor mechanisms and that AUD induces long-lasting changes in HMGB1 signaling. Because the brain is sterile, HMGB1 is a candidate for the endogenous brain TLR agonist that could mediate ethanol induction of TLR receptors in the brain. Ethanol exposure of rodents in vivo increases expression of HMGB1 as well as TLRs 2, 3, 4, and 7 in different brain regions, with associated NF-kappa-B activation and increased cytokines.^15^,^21^,^109^ In vitro culture studies also found that ethanol releases HMGB1 from microglia, astrocytes,^110^ and neurons.^111^ In microglia, ethanol increases expression of TNF-alpha, IL-1-beta, iNOS, and NADPH oxidase.^102^,^103^,^112^ As mentioned above, NF-kappa-B is a key pro-inflammatory transcription factor activated by TLR and cytokines. Ethanol induces NF-kappa-B activation in neurons both in brain slice cultures^21^,^113^ and in vivo^114^ and increases NF-kappa-B binding to DNA,^113^,^115^ consistent with increased transcription of pro-inflammatory cytokines (TNF-alpha, IL-1-beta, and IL-6),^116^ pro-inflammatory oxidases (iNOS),^117^,^118^ cyclooxygenase,^117^,^119^ NADPH oxidase,^102^ and proteases (TNF-alpha-converting enzyme and tissue plasminogen activator).^118^ Astrocyte cell cultures are similarly activated by ethanol.^117^,^120^ In ex vivo brain slice culture, ethanol exposure increases neuroimmune activation,^36^,^118^,^121^ indicating that ethanol can directly cause neuroimmune activation, even in the absence of peripheral immune involvement.

HMGB1-TLR signaling in the brain is a central feature in the neuroimmune responses to ethanol, which contributes to microglial priming. TLR4 was the first TLR discovered, and TLR4 signaling has been studied most extensively in alcohol-induced neuroimmune signaling. Transgenic TLR4 knockout (KO) mice and TLR4 KO glia are protected from neuroimmune activation by ethanol.^117^ This includes protection from glial cell activation, NF-kappa-B activation, and cellular apoptosis indicated by caspase-3 cleavage. Furthermore, TLR4 KO mice are protected from ethanol-induced deficits in anxiety-like behavior and memory impairment, suggesting that TLR4 innate immune signaling is an important mediator of cognitive-behavioral deficits.^112^,^117^,^122^–^124^ In cultured astrocytes, knockdown of TLR4 or TLR adaptor proteins MD-2 or CD14 using small interfering RNA (siRNA) blocks ethanol induction of NF-kappa-B, further suggesting that TLR4 mediates the glial pro-inflammatory response to ethanol.^123^ Alcohol-preferring rats show increased expression of TLR4 in the dopamine reward-mediating ventral tegmental area^125^ that is modulated by GABAA-alpha-2 receptor^57^ and corticotropin-releasing factor.^125^

There are several downstream consequences of TLR4 activation relevant to the progression of drug misuse behaviors. For example, TLR4 activation is also linked to cocaine and heroin use,^126^–^128^ supporting a broader role in addictive pathologies. Although TLR4 is the most often studied TLR in the context of alcohol misuse, several other TLRs are also increased in the postmortem brain of humans with AUD (i.e., TLR2, TLR3, and TLR7) that show pathologic roles in vitro. Thus, TLR2 KO microglia are protected from ethanol-stimulated increases in iNOS and mitogen-activated protein kinases.^112^ Ethanol causes increased expression of TLR3 in neurons, as well as of TLR7 and TLR8 in neurons, astrocytes, and microglial cell lines.^111^ Anti-inflammatory treatments block ethanol-induced increases in brain HMGB1 and reduce alcohol drinking^129^ and other ethanol-induced pathology.^12^,^14^ Further, TLR7 has recently been identified as important in ethanol-induced hippocampal neurodegeneration.^21^ It is possible that initial induction of TLR4 progresses to induction of other TLRs as neuroimmune signaling is amplified by cycles of binge drinking that contribute to the progression across stages of addiction. Although the exact contribution of each TLR across microglia, astrocytes, and neurons remains unclear, it does appear that HMGB1 and multiple TLRs and their immune transcription factors are amplified in the AUD brain and contribute to behavioral pathologies. These studies suggest that ethanol increases HMGB1 and TLR in the brain and contributes to the changes in neurocircuitry related to the development of AUD.

### Adolescent Alcohol Exposure and Risks of AUD

Human studies have indicated that individuals who binge drink in their early teen years have increased levels of lifelong binge drinking and alcohol-related problems, and much higher levels of AUD.^130^–^134^ Adolescent binge drinking also has been linked to long-lasting changes in brain structure and cognitive functioning across a broad range of neuropsychological assessments.^135^ Since human studies have found that adolescent binge drinking changes adult psychology, alcohol drinking, and alcohol-related problems, preclinical models of how adolescent binge drinking alters neuronal networks, and neurobiology can provide insight into mechanisms of brain network adaptation. Human AUD studies, as well as models of adolescent binge drinking and adult AUD, have found increases in brain HMGB1 in multiple regions, although no study has compared brain regions.^14^,^15^,^136^ HMGB1 and TLR signaling crosses microglia, astrocytes, and neurons. Accumulating evidence supports that ethanol-induced changes in microglia, astrocytes, and neurons are related to epigenetic modifications of histone, RNA, and DNA in the nucleus that alter gene expression and cellular phenotypes, which in turn contribute to alterations in neurocircuitry and behavior. For example, studies by the Neurobiology of Adolescent Drinking in Adulthood (NADIA) Consortium using adolescent intermittent ethanol (AIE) exposure to model binge drinking found increases in adult rats’ alcohol drinking, preference for alcohol, as well as anxiety-like behavior. These outcomes all were linked to changes in gene repression signals associated with histone methylation in the central amygdala.^137^,^138^ Reversal of gene silencing mechanisms also reversed anxiety-related behavior as well as increases in adult drinking.^137^,^138^

Other AIE studies found increases in impulsivity, alcohol drinking, and anxiety as well as reversal learning deficits and perseveration.^139^,^140^ Alcohol misuse, anxiety, and impulsivity with continuous thoughts about alcohol and related activities are psychological risk factors for AUD.^139^,^140^ AIE exposure also alters the adult neuronal activation in response to ethanol as indicated by the immediate early response of cFos genes.^141^ AIE-treated rats exposed to acute alcohol as adults have blunted prefrontal cortical ethanol responses and enhanced nucleus accumbens reward pathway responses, consistent with altered neuronal networks that enhance reward responses and reduce prefrontal executive function.^141^ These findings suggest broad AIE-related changes in networks and neurocircuitry.

AIE exposure also adversely impacts other brain regions—notably the hippocampus. The ability of the adult hippocampus to form new neurons (i.e. hippocampal neurogenesis) is reduced following AIE exposure, accelerating the age-related decline of neurogenesis through increases in hippocampal HMGB1 and pro-inflammatory signaling.^142^ Inhibition of pro-inflammatory signaling with exercise or indomethacin prevents and reverses the loss of neurogenesis.^142^–^145^ Interestingly, inhibitors of epigenetic histone deacetylase (HDAC) enzyme, such as trichostatin A (TSA), block LPS-TLR4–induced pro-inflammatory gene induction and microglial responses in the brain.^146^ TSA also reverses AIE exposure-induced anxiety-like and alcohol drinking behaviors, as well as reverses reductions in BDNF mRNA and the histone silencing markers H3K9me2 and H3K27me2 within the BDNF gene in the amygdala and the hippocampus.^147^ Additionally, TSA restores AIE-induced reductions of neurogenesis and hippocampal BDNF levels,^148^ which suggests that TSA inhibition of the microglial pro-inflammatory phenotype is important for the restoration of hippocampal neurogenesis. Finally, TSA inhibits LPS-TLR4 pro-inflammatory signaling and ameliorates pro-inflammatory cognitive changes,^146^ consistent with an increase in HMGB1 acetylation and release resulting from HDAC inhibition^36^ and suggesting that TSA acts directly on cells to reduce pro-inflammatory responses. These studies are consistent with binge drinking-induced HMGB1-TLR pro-inflammatory signaling shifting brain transcription through epigenetic gene expression silencing or enhancing mechanisms that promote persistent increases in pro-inflammatory genes and decreases in trophic-growth factor genes through reversible mechanisms.

Taken together, these findings suggest that multiple pro-inflammatory and TLR targets as well as epigenetic modifications and signaling should be considered as targets for treatment of AUD. The studies indicating reversal across a broad range of binge drinking-induced deficits call for additional studies on how neuronal networks are altered that allow translation to human studies. Further, additional studies are needed to better understand the mechanisms of epigenetic gene silencing, microglial priming, and ethanol pro-inflammatory signaling. Neurotransmitters, peptides, as well as cytokines and HMGB1 relay signals across cells within a brain region that impact cellular responses to ethanol, confounding and complicating generalizations. Across brain regions, however, shifts in cell transcriptomes occur, providing markers of pathology and therapeutic targets.

### Epigenetic Modifications Alter Forebrain Cholinergic Neurons

Neurotransmitters signal across neurons and glia. Acetylcholine is an established neurotransmitter and well-known inhibitor of pro-inflammatory microglial activation through alpha-7 nicotinic receptors.^149^–^151^ Varenicline, which has efficacy in reducing both smoking and alcohol drinking, is a partial alpha-4/beta-2 nicotinic receptor agonist and a full anti-inflammatory nicotinic alpha-7 receptor agonist.^152^ Basal forebrain cholinergic neurons project to the entire cerebrum as well as the hippocampus and contribute to attention, learning, memory, cognitive function, and likely an anti-inflammatory milieu. Their survival and differentiation depend on nerve growth factor. The hippocampus is rich in nicotinic acetylcholine receptors, particularly the alpha-7 subtype found on microglia, and nicotinic agonists block LPS pro-inflammatory signaling.^153^ These studies suggest that cholinergic circuits may also regulate local microglia pro-inflammatory responses. Emerging studies have linked AIE-induced hippocampal pro-inflammatory signaling to loss of neurogenesis that may be driven by the loss of anti-inflammatory cholinergic neuron projections to hippocampal neurogenic regions.^143^ AIE-induced loss of adult basal forebrain cholinergic neurons provides an example of how neurocircuitry is altered by AIE through persistent neuroimmune signaling. Additional studies are needed to determine how many neuronal phenotypes undergo epigenetic shifts during the development of AUD. Loss of neuronal markers has generally been interpreted as loss of neurons; the discovery of reversible epigenetic silencing adds promise to the potential to reverse degeneration.

These studies support the hypothesis that the adolescent brain responds to binge drinking with increases in pro-inflammatory signals involving HMGB1 and TLR that change transcription across neurons, microglia, and astrocytes. Changes in transcription are linked to increases in pro-inflammatory genes and decreases in trophic growth genes through epigenetic gene enhancers or silencers. These changes in cellular signaling, in turn, are linked to alterations in neurocircuits impacting mood, hyperkatifeia symptoms, impulsivity, and perseveration, all of which can promote alcohol drinking and contribute to the development of AUD. This is an oversimplified model of the very complex interactions between numerous factors that are poorly understood; however, further studies on adolescent alcohol-induced loss of cholinergic neurons may provide insight into underlying mechanisms and the potential for treatments.

Forebrain cholinergic neurons are persistently reduced in adulthood following AIE exposure,^52^,^144^ with reductions in hippocampal projections as suggested by loss of hippocampal vesicular acetylcholine transporter^143^ and reduced orbital frontal cortex projections as suggested by blunted microdialysis increases in acetylcholine during a maze challenge.^154^ These findings suggest a retraction of cholinergic projections with reduced forebrain cholinergic neuron cell bodies. Similar to the cortex and the hippocampus, AIE increases HMGB1, TLRs, RAGE, pNF-kappa-B p65 (i.e., phospho-RelA, the phosphorylated p65 that indicates pro-inflammatory signaling), and other neuroimmune genes in the forebrain—changes that persist into adulthood.^16^,^52^,^145^,^155^,^156^ This is associated with reductions in multiple cholinergic genes, including nerve growth factor receptors TrkA and p75NTR, as well as adult cognitive deficits.^52^ Cognitive deficits are linked to changes in forebrain cholinergic neurons that regulate attention and salience essential for learning. Only about a third of the choline acetyltransferase-positive (ChAT+) cholinergic neurons are lost; the remaining ChAT+ neurons are smaller with shrunken soma compared to controls. This loss of ChAT+ cells was initially interpreted as neuronal death. However, there are no indicators of cell death or loss of the global nuclear neuronal marker NeuN, only a loss of cholinergic gene expression and cholinergic neuronal genes.^144^,^157^,^158^ This suggests that AIE suppresses the cholinergic neuronal phenotype, providing insight into a novel mechanism underlying both the persistence and reversibility of ethanol-related changes.^52^

This conclusion is supported by studies showing that inhibition of adult neuroimmune signaling following AIE—for example, by exercise^158^ or treatment with indomethacin^144^ or galantamine^157^—restores cholinergic neurons and reverses learning deficits. Further, cholinergic gene silencing markers, including H3K9me2 and RE-1 silencing element (REST), are induced by AIE and are found on multiple cholinergic genes, consistent with epigenetic silencing mechanisms being reversed with inhibition of neuroimmune signaling and restoration of the cholinergic neurons.^158^ This is further supported by recent forebrain slice culture studies finding that TLR4 signaling through NF-kappa-B induces REST and the gene-silencing methyltransferase G9a, which silences ChAT, TrkA, and other cholinergic genes and thereby shifts neuronal transcription. HMGB1-TLR4-RAGE–induced, ChAT-mediated gene silencing is prevented and reversed by REST knockdown with REST siRNA and silencing histone methyltransferase G9a inhibition.^157^,^158^ These studies suggest that loss of cholinergic neurons is due to loss of cholinergic gene expression (i.e., a loss of cholinergic neuronal phenotype) in some neurons and due to shrinkage of soma in the remaining ChAT+ cholinergic neurons. Studies have found that forebrain cholinergic-GABAergic co-transmission regulates hippocampal responses, and loss of cholinergic synapses, but not GABAergic synapses, interferes with network integration. Cholinergic neurons are known to regulate attention to tasks, likely shifting default mode network (DMN) to prefrontal executive function and salience network (SN). Disruption of these networks following AIE causes a loss of adult behavioral flexibility, such as reversal learning deficits that are restored by anti-inflammatory treatments that also restore cholinergic neurons.^159^,^160^ These findings support pro-inflammatory signaling as mediating reversible epigenetic changes in gene transcription that alter cholinergic neurons and cognition. More studies are needed to better link how transcription shifts link neuronal networks, AUD, and related psychological dysfunction.

### AUD, Neurocircuitry, and Immune Signaling

Understanding the mechanisms of binge drinking-induced changes in brain and behavior requires multiple approaches and a better understanding of adolescent brain, personality, and other developmental processes. Emerging studies of brain physiology have begun to distinguish functional connectivity MRI (fcMRI) networks. There are multiple sensory and motor networks as well as DMN, lateral cortical executive network (CEN), and SN. Although the precise functions of these networks are poorly understood, in general, the CEN engages attention during demanding cognitive tasks and includes the dorsal lateral prefrontal cortex and posterior parietal cortex. CEN activation deactivates the DMN. The DMN—composed of the prefrontal, orbitofrontal, prelimbic, cingulate, retrosplenial, posterior parietal, and temporal association cortex and the dorsal hippocampus—is one of the most robust intrinsic networks that is highly synchronized in the absence of cognitive tasks or saliency.^161^ The SN is a ventral attention network encompassing the anterior insula and cingulate cortex that detects sensory and emotional salient stimuli; it suppresses the DMN and may activate the CEN, consistent with networks directing salient information to arousal and task-solving cortical networks. Imaging studies in humans have identified brain functional network patterns specific to addiction disorders,^162^ and changes in the DMN have been linked to craving and relapse across substance use disorders.^163^ Preclinical studies have found that activation of the locus coeruleus, a key norepinephrine-releasing midbrain nucleus that projects to multiple brain regions, increases DMN connectivity,^164^ whereas stimulation of anterior insular cortical neurons, a key SN node, inhibits the DMN,^165^ supporting the role of these networks as coordinating brain functions. Preclinical studies have identified altered fcMRI network connectivity both in adolescent and adult rats after acute ethanol exposure^166^ and in adult animals long after adolescent AIE binge treatment.^159^,^160^ Acute alcohol exposure increases connectivity within the CEN and connectivity between CEN and DMN as well as CEN and SN. In humans, acute ethanol also increases intra- and inter-network connectivity of the somatosensory cortex.^167^,^168^ Preclinical studies comparing adolescents and adults found that adults are more sensitive to acute binge drinking-induced sedation and fcMRI increases in connectivity than adolescents.^166^ In contrast, adults with prior adolescent AIE show a persistent decrease in adult prefrontal network connectivity.^159^ Preclinical studies also found that treatment with both TLR3^51^,^52^ and TLR756 agonists increased alcohol drinking in operant self-administration paradigms and increased TLR3 and TLR7 expression in the anterior insular cortex, a key hub of the SN.

Additional studies are needed to understand how these network changes relate to AIE-induced increases in drinking, anxiety, and cognitive deficits. Preclinical studies have investigated fcMRI neurocircuits and sickness behaviors (e.g., loss of appetite and motivation, altered mood during infections) following brain pro-inflammatory activation in mice treated with a TLR4 agonist that releases HMGB1 and increases both systemic and brain pro-inflammatory gene expression.^169^ Activation of brain pro-inflammatory signaling by LPS treatment increases brain TNF-alpha, induces sickness behaviors, increases markers of brain microglial activation, and reduces phosphorylated cyclic-AMP response element-binding protein. It also causes a long-term global suppression of network connectivity in regions associated with anhedonia, as well as decreases in DMN connectivity and increases chronic depression-like behavior.^170^ Koob and Volkow have long proposed that SUD and AUD are associated with progressive involvement of brain regions and networks from reward to negative affect, as well as transitioning from impulsive to compulsive drug taking.^1^ Studies reversing TLR increases, increased drinking, and other adaptations need to be linked to changes in fcMRI networks to advance potential targets for therapy and markers of recovery. In summary, neuroimmune TLR signaling may contribute to altered neuronal networks in SUD and AUD.

## Discussion and Conclusions

AUD involves the persistent use of alcohol despite problems, adverse consequences, and harm that occur during drinking to intoxication and that increases in frequency and transitions to preoccupation with alcohol and compulsive harmful alcohol use. AUD development is conceptualized as dysregulation of brain regional networks involving binge drinking, increasing incentive salience, and habitual alcohol drinking frequency. Over several cycles of intoxication and withdrawal, this dysregulation is thought to increase negative emotional states that over time reduce executive functions, goals, and planning and contribute to the compulsive harmful alcohol use of AUD. Koob and Volkow^1^ have proposed that several neurocircuits are involved in these functional domains, which overlap with networks identified by fcMRI as the SN, CEN, and DMN. Whereas acute ethanol consumption increases connectivity within these networks, chronic AIE studies found reduced connectivity consistent with adaptations during and after alcohol drinking; however, the relationship of these adaptations with AUD symptoms is poorly understood. Changes in these networks, particularly the DMN, have been linked to SUD and AUD and to predicting relapse in people recovering from these disorders; these changes are complex and involve various subnetworks.

Other studies found that these networks are also altered by brain pro-inflammatory gene induction. Substantial literature supports pro-inflammatory gene induction in the brain as contributing to depressive disorder and negative affect, which overlaps with hyperkatifeia and other negative emotional psychological adaptations found during chronic ethanol misuse. The amygdala, anterior insula, and anterior cingulate cortex, which are part of the SN, are linked to emotional responses and connectivity that is disrupted in individuals with depression, consistent with a role for the SN in negative affect. In preclinical studies, pro-inflammatory brain activation suppressed network connectivity, although more studies need to be done to understand these mechanisms. The studies reviewed here suggest that binge drinking increases HMGB1-TLR pro-inflammatory signaling that leads to altered cellular transcriptomes and changes in synapses and disrupted networks through epigenetic mechanisms.

Although induction of pro-inflammatory signaling resulting from binge drinking can persist for long periods in the brain, it can be reversed. As reviewed here, pro-inflammatory signaling is linked to AUD, suggesting a role for anti-inflammatory treatments, although more studies are needed. Indomethicin is a particularly strong anti-inflammatory that protected against binge drinking pathology in preclinical studies. Other neuroimmune treatment strategies for AUD are being tested.^4^,^12^ Glia and neurons express pro-inflammatory genes, and microglial depletion studies have shown that microglia regulate mouse escalation of drinking.^171^,^172^ Similarly, minocycline, an antibiotic that also inhibits microglial function,^173^ reduces rat ethanol self-administration in vivo.^174^ Phosphodiesterase 4 (PDE4) inhibitors, which exert anti-inflammatory actions via NF-kappa-B inhibition, presumably through a cAMP-mediated mechanism,^175^ have also been found to reduce ethanol self-administration in vivo.^176^ For example, the PDE4 inhibitors apremilast and rolipram reduce alcohol consumption and alter acute responses to alcohol in preclinical studies.^177^–^179^ The PDE4 inhibitor ibudiblast may have efficacy in AUD,^4^ but preclinical studies suggested it does not reduce ethanol-induced pro-inflammatory responses.^93^ Allopregnanolone, a neurosteroid known to reduce alcohol drinking, was discovered to block TLR4 receptors, thereby blocking ethanol activation of pro-inflammatory signaling^180^ and reducing alcohol drinking; this is being further investigated through clinical trials. These findings are related to general anti-inflammatory effects and are not specifically focused on HMGB1; however, they are consistent with persistently increased HMGB1 neuroimmune signaling playing a role in altering networks. Many of these preclinical studies found that inhibition of neuroimmune signaling restores binge drinking-induced adaptations in HMGB1, TLR, and other pro-inflammatory genes; loss of neurogenesis and cholinergic neurons; and cognitive deficits and anxiety. Although studies have found that anti-inflammatory medications can reduce binge drinking and reverse chronic alcohol-induced brain pathology, additional studies are needed to fully understand how changes in pro-inflammatory and other gene transcription can lead to adaptations in psychological factors, and how they are restored by therapies.

**Figure 3:**
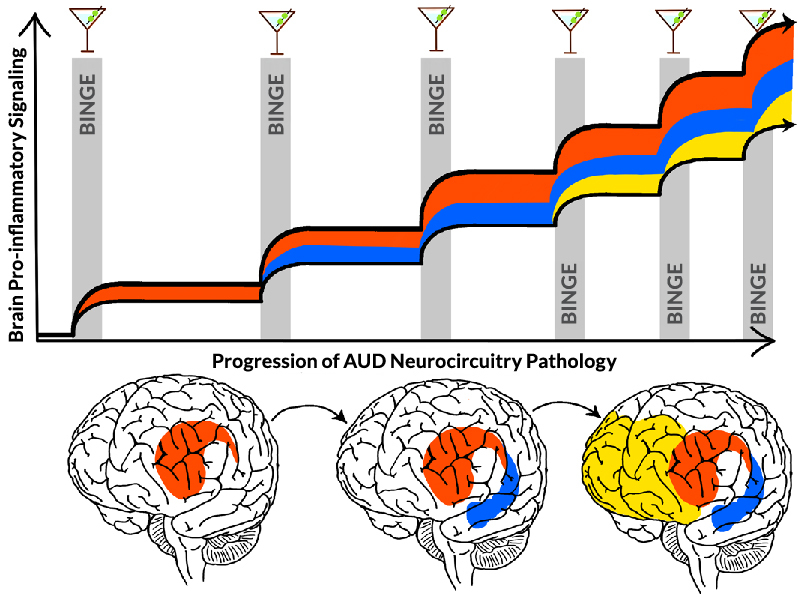
Hypothetic mechanism of cycles of binge drinking intoxication increasing pro-inflammatory gene transcription to increasingly compromise neuronal networks that drive the progression to alcohol use disorder (AUD) With each binge drinking event (gray bars), pro-inflammatory gene induction and transcription changes increase (adapted from Koob and Volkow^1^). Initial stages of binge drinking increase HMGB1 release and other signals that sensitize microglia and activate reward and emotional salience networks (red). This activation spreads with further cycles, progressively increasing involvement of emotional-salience networks (blue) as binge drinking increases in frequency. Further cycles may increase pro-inflammatory signaling that compromises cortical executive function networks (yellow). Together, these networks affect domains associated with reward seeking, impulse inhibition, perseveration, and compulsion to drink that occur with AUD. It is unknown, however, whether networks become progressively involved with binge-drinking cycles as depicted here or if each network shows accumulation of pro-inflammatory and network dysfunction with cycles. *Note:* HMGB1, high mobility group box protein 1.

This review proposes that HMGB1-TLR pro-inflammatory signaling induces epigenetic gene silencing and enhancing mechanisms that alter glial and neuronal transcriptomes, changing cellular phenotypes. Evidence supports that epigenetic gene-regulating mechanisms such as histone methylation are involved in causing microglial and astrocyte sensitization or priming that increases pro-inflammatory gene expression, including TLR and other genes. Further, studies found that cholinergic neurons respond to HMGB1-TLR signaling with increases in REST that silence ChAT and other cholinergic genes, changing neuronal phenotypes (i.e., reducing cholinergic neurotransmission). These epigenetic shifts in cellular phenotype are proposed to contribute to altered networks that show progressive involvement of brain regions, consistent with the progressive changes across binge intoxication, negative affect, and cognitive dysfunction components of AUD (Figure 3). Inhibitors of epigenetic signals, such as anti-inflammatory treatments, can reverse and prevent binge drinking adaptations across glia and neurons, as well as behavioral changes in alcohol drinking, anxiety, and cognition. The proposal that components of AUD are due to epigenetic-pro-inflammatory mechanisms is consistent with reversal of binge drinking pathology in microglia, astrocytes, and neurons. This offers great promise for reversal of AUD pathology, which is often considered permanent. Emerging neuroimmune-epigenetic signaling studies provide hope that chronic relapsing AUD may be reversed through these newly discovered mechanisms. The mechanisms proposed in this review support therapeutic targets that could reverse both cellular phenotypes and AUD psychological phenotypes; they also provide a framework from brain cellular biology to neuronal networks to AUD pathology that hopefully will advance efforts to understand, prevent, and treat AUD.
